# Association of Child and Family Attributes With Outcomes in Children With Autism

**DOI:** 10.1001/jamanetworkopen.2021.2530

**Published:** 2021-03-29

**Authors:** Peter Szatmari, Katherine Tombeau Cost, Eric Duku, Teresa Bennett, Mayada Elsabbagh, Stelios Georgiades, Connor Kerns, Pat Mirenda, Isabel M. Smith, Wendy J. Ungar, Tracey Vaillancourt, Charlotte Waddell, Anat Zaidman-Zait, Lonnie Zwaigenbaum

**Affiliations:** 1 The Hospital for Sick Children, Toronto, Ontario, Canada; 2 Centre for Addiction and Mental Health, Toronto, Ontario, Canada; 3 McMaster University, Hamilton, Ontario, Canada; 4 McGill University, Montreal, Quebec, Canada; 5 University of British Columbia, Vancouver, British Columbia, Canada; 6 Dalhousie University, Halifax, Nova Scotia, Canada; 7 University of Ottawa, Ottawa, Ontario, Canada; 8 Simon Fraser University, Vancouver, British Columbia, Canada; 9 Tel Aviv University, Tel Aviv, Israel; 10 University of Alberta, Edmonton, Alberta, Canada

## Abstract

**Importance:**

The prevalence and attributes of positive outcomes (or *doing well*) among children with autism spectrum disorder (ASD) in midchildhood are not well known.

**Objective:**

To estimate the prevalence of doing well according to metrics of proficiency and growth and to investigate the extent to which significant associations exist between child- and family-level variables and doing well.

**Design, Setting, and Participants:**

This longitudinal cohort study included children with ASD from regional clinics across Canada. Participants were sampled 3 times between ages 2 and 4.9 years (T1) and twice in follow-up into middle childhood (T2). Data were analyzed March 2018 through January 2020.

**Exposures:**

Language and IQ assessments at first sample; household income, parent coping, and family functioning.

**Main Outcomes and Measures:**

Key outcome domains of developmental health included measures of socialization, communication, independent living skills, and measures of internalizing and externalizing behaviors. Thresholds for doing well in these domains by either proficiency or growth were established. The extent to which language, IQ, household income, parent coping, and family functioning were associated with assessed outcomes was determined by logistic regression. The association between outcomes and concurrent Autism Diagnostic Observation Schedule (ADOS) classification scores was also estimated.

**Results:**

In a total cohort of 272 children (234 [86.0%] boys; mean [SD] age, 10.76 [0.26] years), approximately 78.8% (95% CI, 73.2%-84.4%) of the sample were estimated to be doing well by either metric on at least 1 domain, and 23.6% (95% CI, 17.7%-29.4%) were doing well in 4 or 5 domains. It was possible to be doing well by either proficiency or growth and still meet ADOS criteria for ASD. For the growth metric, between 61.5% (95% CI, 40.7%-79.1%) and 79.6% (95% CI, 66.0%-88.9%) of participants had ADOS scores of 4 or greater; for the proficiency metric, between 63.8% (95% CI, 48.4%-76.9%) and 75.8% (95% CI, 63.0%-85.4%) had scores of 4 or greater. Doing well by either metric for all domains was associated with T1 scores on that outcome domain (eg, T1 daily living skills associated with doing well at T2 daily living by the proficiency metric as measured by the Vineland Adaptive Behavior Scales–Second Edition daily living skills scale [202 participants]: β = 0.07; OR, 1.07; 95% CI, 1.03-1.11; *P* < .001). Doing well in socialization by the growth metric was also associated with better T1 language skills scores (202 participants) (β = 0.04; OR, 1.04; 95% CI, 1.00-1.07, *P* = .04). Doing well in externalizing by the growth metric was also associated with higher household income at T1 (178 participants) (β = 0.10; OR, 1.10; 95% CI, 1.06-1.15; *P* < .001). Better family functioning at T1 was associated with doing well on both socialization and externalizing by proficiency metric and on internalizing by growth metric (socialization by proficiency [202 participants]: β = −1.01; OR, 0.36; 95% CI, 0.14-0.93; *P* = .04; externalizing by proficiency [178 participants]: β = 1.00; OR, 0.37; 95% CI, 0.16-0.82; *P* = .02; internalizing by growth [178 participants]: β = −1.03; OR, 0.36; 95% CI, 0.16-0.79; *P* = .01).

**Conclusions and Relevance:**

This cohort study found that a substantial proportion of children with ASD were doing well by middle childhood in at least 1 key domain of developmental health, and that doing well was possible even in the context of continuing to meet criteria for ASD. These results support a strengths-based approach to treatment planning that should include robust support for families to increase the potential likelihood of doing well later in life.

## Key Points

**Question:**

How prevalent is “doing well” in 5 developmental domains (communication, socialization, activities of daily living, internalizing, externalizing) as assessed by proficiency and growth in children with autism spectrum disorder in midchildhood, and what attributes of children and families are associated with it?

**Findings:**

In this cohort study including 272 children, between 20% and 49% of children with autism spectrum disorder were proficient within the 5 developmental domains, while 13% to 34% of children demonstrated growth. Doing well was associated with preschool scores on that specific outcome domain, as well as early language skills, household income, and family functioning.

**Meaning:**

These findings demonstrate the potential usefulness of taking a strengths-based approach to outcome assessments, while the importance of family income and functioning remind us that disabilities do not exist apart from a social context.

## Teaser

This cohort study of children with autism spectrum disorder in Canada assesses the prevalence of positive developmental outcomes using clinical metrics of proficiency and growth and examines associations between outcomes and individual- and family-level variables.

## Introduction

Autism spectrum disorder (ASD) is a lifelong condition affecting multiple developmental domains.^1^ Pathways in ASD^2^ study researchers held conferences over several years with parents, youth, practitioners, policy makers, and researchers to formulate key evidence gaps that should be addressed by the field. Through these conferences, the group reached consensus that the most important evidence gap in ASD for a Canadian context was, “What factors are associated with good outcomes in ASD?”

This question, to some extent, has been addressed in systematic reviews and meta-analyses of adult outcomes.^3,4^ In primary studies, investigators used a common outcome tool^5,6^ combining metrics of attainment of emerging adult milestones in socialization, occupational status, and independent living into 1 score. Adults were stratified into groups ranging from good to poor outcomes. In a 2020 meta-analysis,^7^ the pooled estimate of a good adult outcome was 20% with substantial heterogeneity between studies.^4^

The definition of a good outcome for those with ASD at different developmental stages, such as late childhood or adolescence, is less clear. Recent longitudinal follow-up studies in childhood and adolescence have taken a person-centered approach to focus on individual differences in development in people with ASD^8,9^ or else have used intervention studies to measure relatively short-term outcomes.^10^ Indeed, much of the literature in this area takes a deficit-based measurement approach of good, best, or optimal outcomes that focuses on an absence of intellectual disability and no longer meeting criteria for ASD.^11,12^ While this approach has some face validity and has proved enlightening, a more nuanced and holistic approach might provide a more complete picture of outcomes generally (as argued by Mason et al^7^).

The definition of a good outcome is somewhat more ambiguous now than previously thought. The neurodiversity movement and involvement of those with lived experience^13^ in advocacy and research have provided much-needed perspectives. Given that ASD is multidimensional and heterogeneous, the developmental domains that should be considered as outcomes and the measurement tools that should be used to assess those domains is an evolving issue.^14^ Furthermore, consensus is still needed on criteria for defining a good outcome. For example, many individuals with lived experience^15^ would consider themselves as having a good outcome^16,17^ despite—or because of—retaining an ASD diagnosis. McCauley et al^18^ have adopted a novel approach in line with this view with their definition of different types of positive outcomes in adulthood depending on cognitive ability.

For this developmental stage, we prefer the phrase “doing well,” a less value-laden concept than having a good outcome. Specifying an outcome implies a final endpoint, whereas doing well relates to someone’s circumstances at a particular point in their life’s journey. A measurement framework for doing well that can be applied at multiple points in time is important. Taylor^19^ has outlined 2 possible ways to define doing well in ASD in general terms that address some of the above concerns: (1) “proficiency” sets a specific threshold of competence to be met, regardless of the starting point, whereas (2) “growth” sets a specific amount of improvement between 2 time points. In this sense, a child with ASD can be doing well compared with typically developing children by reaching a level of proficiency, or can be doing well by showing growth. Among other advantages, this approach can be flexibly applied to different developmental domains, measurement tools, developmental stages, and to children with diverse abilities.

Our objective with this study was to apply Taylor’s^19^ framework of growth and proficiency to children with ASD in middle childhood. Our primary research questions were: (1) What is the prevalence of doing well? and (2) What child- and family-level attributes are associated with doing well in midchildhood? We selected multiple outcome domains based on parent input and the literature. Given the paucity of research on family and contextual factors that might be associated with outcomes in ASD,^20^ we chose attributes based on the literature or on variables that were potentially modifiable.

## Methods

### Participants

Recruitment occurred through ASD clinics in Halifax, Montreal, Hamilton, Edmonton, and Vancouver, Canada. Each center provided diagnostic services to nearly all young children suspected of having ASD within administratively defined geographic regions, which included urban and rural communities surrounding those cities. An inception cohort of consecutive cases included children aged 2 to 4.9 years, diagnosed with ASD by agreement between clinical opinion, the Autism Diagnostic Interview–Revised^21^ (ADI-R), and the Autism Diagnostic Observation Schedule^22^ (ADOS). Children included in the cohort had an estimated nonverbal mental age of at least 18 months (the minimum for the ADI-R). Exclusion criteria were genetic disorders, cerebral palsy, blindness, and deafness. One child per family was invited to participate. Children were enrolled^23^ at, or soon after, diagnosis (mean [SD] enrollment, 2.7 [2.5] months after diagnosis), at a mean (SD) age of 3.4 (0.8) years.

Information was drawn from 5 data points in the Pathways data set (see eFigure in the Supplement). Time 1 (T1) was composed of 3 data points during early childhood (mean [SD] ages of 3.4 [0.8], 4.0 [0.8], and 4.5 [0.8] years). Data collection for T1 began in May 2005 and ended in October 2012. Time 2 (T2) was composed of 2 data points during middle childhood (mean [SD] ages of 8.7 [0.2] and 10.8 [0.3] years). Data collection for T2 began in May 2009 and continued through March 2018. At T2, 64.6% of the original sample was retained and assessed. (Sample characteristics are available in Table 1.) Assessments typically took place in clinics and research centers, with some conducted in community settings and homes. The study was reviewed and approved by all local research ethics boards and informed consent was obtained in writing by all families. Data analyses were completed March 2018 through January 2020. We adhered to the Strengthening the Reporting of Observational Studies in Epidemiology (STROBE) reporting guideline for cohort studies.

**Table 1.** Characteristics of Participants

Characteristics	T1		T2	
	Participants, No. (N = 272)	Mean (SD)	Participants, No. (N = 272)	Mean (SD)
Age, mo	272	40.73 (8.95)	198	129.08 (3.10)
Boys, No. (%)	272	234 (86.0)	272	234 (86.0)
Girls, No. (%)	272	38 (14.0)	272	38 (14.0)
Developmental domain				
VABS-II Communication	272	74.59 (16.26)	272	79.52 (16.39)
VABS-II Activities of daily living	272	76.08 (11.42)	272	77.26 (12.56)
VABS-II Socialization	272	72.20 (9.12)	272	73.86 (13.61)
CBCL Internalizing	255	60.29 (9.25)	228	53.96 (10.17)
CBCL Externalizing	255	56.52 (10.44)	228	51.06 (10.81)
Covariates				
Merrill-Palmer	237	58.51 (25.85)	NR	NR
PLS-4	262	66.74 (19.50)	NR	NR
Ways of coping	262	0 (0.96)	NR	NR
ADOS	271	7.68 (1.68)	202	6.81 (2.62)
General family functioning	260	1.75 (0.47)	NR	NR
Socioeconomic, No. (%)				
≥Bachelor’s degree attained by PMK	260	108 (41.6)	172	68 (39.6)
≥Bachelor’s degree attained by partner	248	91 (36.7)	154	48 (31.1)
Married or common law	262	242 (92.4)	172	146 (84.9)
Full-time employment of PMK	263	76 (28.9)	171	72 (42.1)
Full-time employment of partner	251	209 (83.3)	154	126 (81.9)
Income >$80 000 per year	256	102 (39.8)	169	86 (50.9)

Abbreviations: ADOS, Autistic Diagnostic Observation Schedule; CBCL, Child Behavior Checklist; NR, not reported; PLS-4, Preschool Language Scale, Fourth Edition; PMK, Person Most Knowledgeable; VABS-II, Vineland Adaptive Behavior Scales–Second Edition.

### Procedure

#### Outcome Domain Selection

An advisory group of parents identified areas, such as peer relationships, communication, emotional health, and independent living skills, as outcomes important to them. These domains were very similar to domains identified in a comprehensive scoping review with stakeholder input of outcomes for younger children.^24^ We selected standardized measures from our assessment battery that matched these constructs and for which cutoffs could be derived to identify those who were doing well relative to a neurotypical population.

### Instruments

#### Outcome Measures

Peer relationships, communication, and independent living skills were assessed using the Vineland Adaptive Behavior Scales–Second Edition (VABS-II) socialization, communication, and daily living skills scales.^25^ The VABS-II is a semistructured interview measuring adaptive behavior with standard scores (*M* = 100, SD = 15). We defined proficiency as a standard score 85 or above (ie, 1 SD below the mean). This metric for “not impaired” was used in other optimal outcome studies^26^ and in the learning disability literature. We defined growth as improvement of at least 1 SD in standard scores (15 points) from T1 to T2. Improvement of 1 SD in clinical trials is considered a large effect size.^27,28,29^

Emotional health was assessed using the Child Behavior Checklist^30^ (CBCL). The CBCL for Ages 1.5-5 at T1 and the CBCL for Ages 6-18 at T2 was completed by a primary caregiver (usually the mother) based on observations of the child’s behavior in the 2 months prior. The CBCL yields 2 composite behavior scores: internalizing and externalizing. The CBCL has excellent psychometric properties^30^ and has been widely used in this population.^31,32^ Cut points of 70 or 60 or below may still represent subthreshold psychopathology in this population,^33,34^ so proficiency is defined as a T score of 50 or below (the population mean) for a more conservative estimate. We defined growth as improvement (ie, a T score decrease) of at least 1 SD (10 points) between T1 and T2.

#### T1 Attribute Measures

We selected T1 cognitive and language measures as traditional within-child attributes. General cognitive functioning was assessed with the Merrill-Palmer-Revised.^35^ We used the Developmental Index standard score, comprising cognitive, receptive language, and fine motor scales. Language skills at T1 were assessed with the Preschool Language Scale, Fourth Edition,^36^ a comprehensive language test reflecting receptive and expressive language abilities.

Based on previous work,^37,38^ we included 3 contextual variables completed by the primary caregiver: household income, parent coping, and family functioning. Household income was dichotomized as less than $80 000 per year and $80 000 per year or more based on a bimodal distribution in our sample. Parent disengaged emotion-focused coping was assessed with the Ways of Coping questionnaire.^39^ Disengaged emotion-focused coping comprises items related to parents’ attempts to distance themselves, avoid, or minimize a stressor and negative emotions associated with a stressor.^37^ Family functioning was assessed with the general family functioning subscale of the McMaster Family Assessment Device.^40^ This tool assesses the characteristics of the family regarding communication, discipline, and support among family members.

#### ADOS Classification at T2

The ADOS^22^ is a semistructured, standardized observational measure that assesses social and communication behavior indicative of ASD, administered by research-reliable examiners at each site. We used the calibrated severity metric^41^ with scores ranging from 1 to 10 at T2. A score of 1 to 3 is classified as non-ASD, whereas a score of 4 or more is classified as consistent with ASD.

#### SRS Classification at T2

The Social Responsiveness Scale (SRS) teacher report is a questionnaire completed by a child’s teacher that assesses social impairment indicative of ASD.^42^ A score of 60 or less is classified as non-ASD, whereas a score greater than 60 is classified as consistent with ASD.^42^

### Statistical Analysis

#### Missing Data

We compared participants with missing data with those with complete data on the variables of interest. Differences between participants who completed only T1 measures compared with participants who completed both T1 and T2 measures are in eTable 1 in the Supplement.

#### Attribute and Outcome Variables

For variables that were collected more than once within the T1 or T2 spans, we used mean scores to obtain a more stable assessment (see eFigure in the Supplement). In all cases, identical measures used within either the T1 span (3 data collection points within T1) or the T2 span (2 data collection points within T2) were correlated (*P* < .001, *r* > .55; see eTable 2 in the Supplement). Furthermore, scores were stable within individuals based on *z*-transformed SDs across scores for each participant below an a priori threshold of 3 SDs (with all SDs being between 0.00 and 2.42), which was used to identify outlier participants with respect to change between combined time points.

#### Measurement Model

The growth and proficiency criteria for doing well were applied to each of the 5 outcome domains. To assess the measurement model, we determined the extent to which doing well was a multivariable concept composed of distinct metrics. Agreement between and within the proficiency and growth metrics was evaluated with Cohen κ. The threshold for distinct measures (ie, lack of agreement between measures) was 0.40, less than what is considered moderate to strong agreement.^43^

#### Prevalence of Doing Well by Domain

For each domain, we created a 4-level classification outcome variable of doing well based on: (1) only the growth criterion, (2) only the proficiency criterion, (3) both criteria, or (4) neither criterion. We estimated the prevalence and 95% confidence intervals of each of these outcome classifications for each domain. To estimate the extent to which doing well was similar across multiple domains within children, we calculated the number of domains in which a child was assessed to be doing well at T2 by any metric.

#### Association Between Doing Well and ADOS and SRS Classification

We determined the proportion of children identified as having growth or proficiency in the various domains who scored above and below the cutoff for ASD on the ADOS at T2 and those who scored above and below the cutoff on the teacher SRS at T2. The agreement between classification on the ADOS or SRS and doing well was estimated using Cohen κ; under 0.40 was considered weak agreement.

#### Attributes of Doing Well in Specific Domains

We hypothesized that better child cognitive and language skills, higher family income, better family functioning, and less use of emotion-focused coping would be associated with doing well in middle childhood. The analyses were done separately for each domain within each metric. Six attributes were entered into binary logistic regression, controlling for the T1 domain-specific ability. The Hosmer-Lemeshow test was used to estimate model fit. To control for type I error, we applied a Bonferroni correction across the 10 omnibus logistic regressions (α = .005). Given a significant omnibus test, 2-sided *P* < .05 determined significance and odds ratios (ORs) for the post hoc tests. Data were analyzed using SPSS version 24.0 (IBM) and R version 3.6 (R Project for Statistical Computing).

## Results

The total sample of 272 children was composed of 234 boys (86.0%), and had a mean (SD) age of 3.39 (0.75) years at T1 and 10.76 (0.26) years at T2 (Table 1). Participants missing T2 data had lower VABS-II Communication and PLS scores at T1 than those with complete data and more frequently had a lone parent or parents living in a common-law relationship (eTable 1 in the Supplement). Sample size was not limited by T1 domain data. To reduce bias because of missing not at random in a regression model,^44^ we did not impute data.^45^ The sample size for each analysis is therefore provided with the results of each analysis.

### Measurement Model

Agreement was only moderate between the different measures of doing well by the proficiency metrics within instruments: VABS-II (275 total respondents; κ = 0.30-0.57) and CBCL (224 total respondents; κ = 0.45-0.48; eTable 3 in the Supplement). Agreement between VABS-II and CBCL was subthreshold (213 total respondents; κ = 0.04-0.29). There was moderate agreement between metrics of growth and proficiency within a domain (κ = 0.29-0.55; eTable 3 in the Supplement). For the most part, then, the different measures of doing well are distinct.

### Prevalence of Doing Well by Domain

By middle childhood, 20.2% of participants were proficient (ie, scoring in the not impaired range) in socialization (55 of 272 participants; 95% CI, 15.7%-25.6%), 24.3% in daily living skills (66 of 272 participants; 95% CI, 19.4%-29.9%), and 38.2% in communication (104 of 272 participants; 95% CI, 32.5%-44.3%). In CBCL domains, 35.9% were proficient for internalizing behavior (79 of 220 participants; 95% CI, 29.6%-42.7%), whereas 49.1% were proficient on externalizing problems (108 of 220 participants; 95% CI, 42.3%-55.9%). Using the growth criterion, doing well ranged from 12.5% in daily living skills (34 of 272 participants; 95% CI, 8.9%-17.2%) to 34.5% for internalizing problems (76 of 220 participants; 95% CI, 28.4%-41.3%) (Figure). A total of 78.8% (168 of 213 participants; 95% CI, 73.2%-84.4%) of the sample were doing well by either metric on at least 1 domain, and 23.6% (50 of 213 participants; 95% CI, 17.7%-29.4%) were doing well in 4 or 5 domains (Figure).

**Figure.**
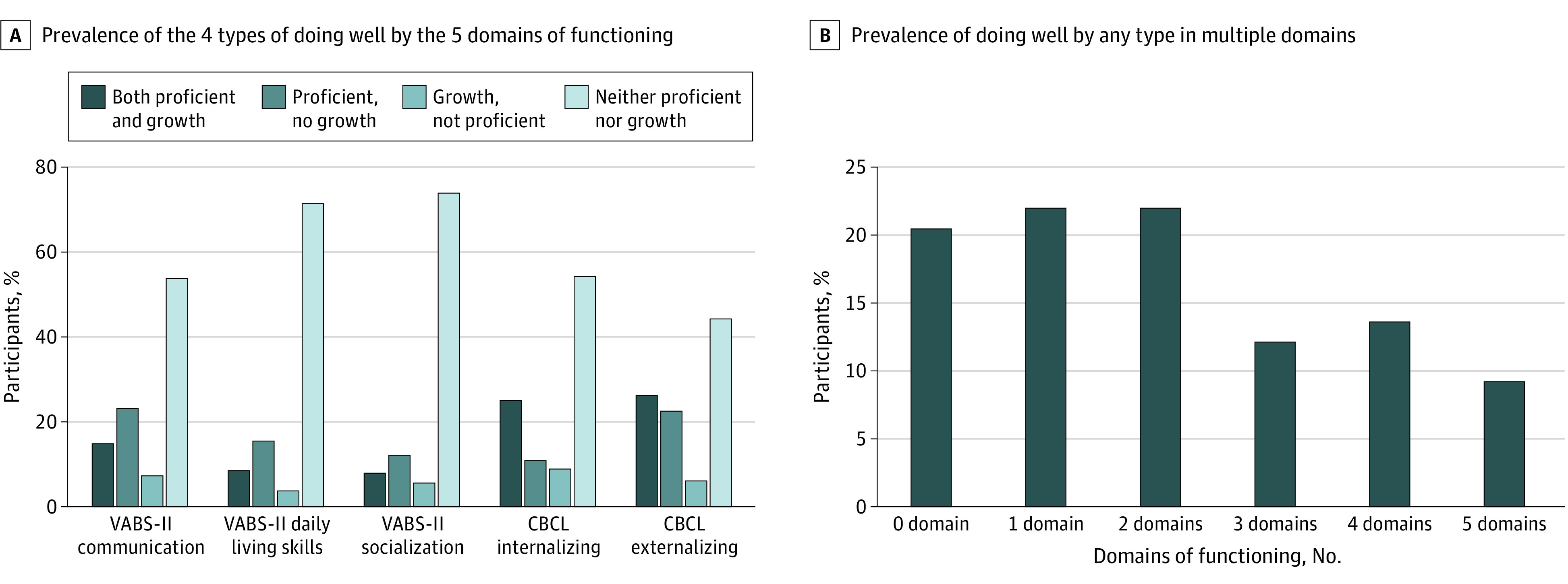
Outcomes Among Children With Autism Spectrum Disorder Measured by Proficiency and Growth CBCL indicates Child Behavior Checklist; VABS-II, Vineland Adaptive Behavior Scales–Second Edition.

### Prevalence of Doing Well Compared With ADOS or SRS Classification

Some children were doing well in specific domains and still scoring above the ASD cutoff on the ADOS (Table 2 and Table 3). For the growth metric, between 61.5% (95% CI, 40.7%-79.1%) and 79.6% (95% CI, 66.0%-88.9%) of participants had ADOS scores of 4 or greater; for the proficiency metric, between 63.8% (95% CI, 48.4%-76.9%) and 75.8% (95% CI, 63.0%-85.4%) had scores of 4 or greater. Cohen κ were consistently below the 0.40 threshold, suggesting little agreement between meeting ASD criteria on the ADOS and doing well by either metric in any domain. All Cohen κ were consistently below threshold, again indicating little agreement on meeting ASD criteria on the teacher-scored SRS and doing well by either metric (VABS-II domains, 139 participants; CBCL domains, 122 participants) (eTable 4 in the Supplement).

**Table 2.** Participants With ADOS Scores <4 or ≥4 for Growth Outcome Metric Within Each Domain

Domain	Growth			No growth		
	No.	ADOS <4, % (95% CI)	ADOS ≥4, % (95% CI)	No.	ADOS <4, % (95% CI)	ADOS ≥4, % (95% CI)
VABS-II (n = 202)						
Communication	44	22.7 (12.0-38.2)	77.3 (61.8-88.0)	158	14.6 (9.7-21.3)	85.4 (78.8-90.3)
Socialization	26	38.5 (20.9-59.3)	61.5 (40.7-79.1)	176	13.1 (8.7-19.2)	86.9 (80.8-91.3)
Activities of daily living	23	30.4 (14.0-53.0)	69.6 (47.0-86.0)	179	14.5 (9.9-20.7)	85.5 (79.3-90.1)
CBCL (n = 170)						
Internalizing	59	22.0 (12.7-35.0)	78.0 (65.0-87.3)	111	15.3 (9.4-23.7)	84.7 (76.3-90.6)
Externalizing	54	20.4 (11.1-34.0)	79.6 (66.0-88.9)	116	16.4 (10.4-24.7)	83.6 (75.3-89.6)

Abbreviations: ADOS, Autistic Diagnostic Observation Schedule; CBCL, Child Behavior Checklist; VABS-II, Vineland Adaptive Behavior Scales–Second Edition.

**Table 3.** Participants With ADOS Scores <4 or ≥4 for Proficiency Outcome Metric Within Each Domain

Domain	Proficient			Not proficient		
	No.	ADOS <4, % (95% CI)	ADOS ≥4, % (95% CI)	No.	ADOS <4, % (95% CI)	ADOS ≥4, % (95% CI)
VABS-II (n = 202)						
Communication	83	27.7 (18.7-38.3)	72.3 (61.2-81.3)	119	8.4 (4.3-15.3)	91.6 (84.7-95.7)
Socialization	47	36.2 (23.1-51.6)	63.8 (48.4-76.9)	155	10.3 (6.2-16.5)	89.7 (83.5-93.8)
Activities of daily living	53	30.2 (18.7-44.5)	69.8 (55.5-81.3)	149	11.4 (7.0-17.9)	87.9 (81.3-92.5)
CBCL (n = 170)						
Internalizing	62	24.2 (14.6-37.0)	75.8 (63.0-85.4)	108	13.9 (8.2-22.2)	86.1 (77.8-91.8)
Externalizing	83	25.3 (16.7-36.2)	74.7 (63.8-83.3)	87	10.3 (5.1-19.1)	89.7 (80.9-94.9)

Abbreviations: ADOS, Autistic Diagnostic Observation Schedule; CBCL, Child Behavior Checklist; VABS-II, Vineland Adaptive–Second Edition.

### Attributes of Doing Well in Specific Domains

The omnibus test was significant for associations with proficiency in all domains except CBCL Internalizing (eTable 5 in the Supplement). In post hoc logistic regression for proficiency in each of these domains, the T1 score in the outcome domain was positively associated with the outcome. That is, a high T1 score on the VABS-II or a low T1 score on the CBCL was associated with a proficient outcome at T2 (T1 communication [202 participants]: β = 0.05; OR, 1.05; 95% CI, 1.01-1.09; *P* = .01; T1 daily living skills [202 participants]: β = 0.07; OR, 1.07; 95% CI, 1.03-1.11; *P* < .001; T1 socialization [202 participants]: β = 0.10; OR, 1.11; 95% CI, 1.06-1.16; *P* < .001; T1 externalizing [178 participants]: β, = –0.06; OR, 0.94; 95% CI, 0.91-0.98; *P* = .001). The only other variable that was significantly associated with doing well by the proficiency metric was general family functioning, for both externalizing (β = 1.00; OR, 0.37; 95% CI, 0.16-0.82; *P* = .02) and socialization (β = –1.01; OR, 0.36; 95% CI, 0.14-0.93; *P* = .04) (Table 4).

**Table 4.** T1 Attributes Associated With T2 Doing Well by the Proficient Metric in Each of the 5 Domains

Characteristic	OR (95% CI)				
	VABS-II Communication	VABS-II Activities of daily living	VABS-II Socialization	CBCL Internalizing^a^	CBCL Externalizing
T1 instrument score^b^	1.05 (1.01-1.09)^c^	1.07 (1.03-1.11)^c^	1.11 (1.06-1.16)^c^	0.96 (0.92-1.00)	0.94 (0.91-0.98)^c^
SES^d^	0.8 (0.40-1.58)	0.64 (0.32-1.28)	1.41 (0.64-3.10)	0.82 (0.42-1.58)	0.54 (0.28-1.08)
Sex^e^	0.86 (0.31-2.41)	1.76 (0.61-5.13)	1.22 (0.39-3.86)	0.64 (0.25-1.60)	1.8 (0.69-4.73)
Merrill Palmer	1.02 (0.99-1.04)	1 (0.97-1.02)	1.02 (0.99-1.04)	0.99 (0.96-1.01)	0.99 (0.96-1.01)
PLS-4	1.02 (0.98-1.05)	1.03 (1.00-1.06)	1.01 (0.98-1.04)	1.01 (0.98-1.04)	1.02 (0.99-1.06)
Ways of coping	0.38 (0.13-1.09)	0.52 (0.18-1.54)	0.68 (0.22-2.15)	1 (0.36-2.74)	0.38 (0.13-1.07)
General family functioning	0.84 (0.38-1.85)	0.59 (0.26-1.33)	0.36 (0.14-0.93)^c^	0.42 (0.19-0.93)	0.37 (0.16-0.82)^c^

Abbreviations: CBCL, Child Behavior Checklist; OR, odds ratio; PLS-4, Preschool Language Scale, Fourth Edition; SES, socioeconomic status; T1, samples assessed between ages 2 and 4.9 years; T2, samples assessed in middle childhood; VABS-II, Vineland Adaptive Behavior Scales–Second Edition.

^a^ The omnibus test for this model was not significant and the results on variables associated with this outcome should not be interpreted (eTable 5 in the Supplement).

^b^ T1 instrument scores represent the score on the instrument at T1 in the domain being analyzed (ie, in the analysis on VABS-II Communication outcome at T2, the instrument score VABS-II Communication at T1 is included in analysis).

^c^ *P* < .05.

^d^ Estimated self-reported income stratified at $80 000, with 0 = <$80 000 per year household income, and 1 = ≥$80 000 per year household income.

^e^ Sex coded with 0 = male and 1 = female (ie, an OR > 0 indicates a female advantage).

In investigating associations between attributes and doing well by the growth metrics, the omnibus test was significant for growth in all domains except daily living skills (Table 5). In post hoc logistic regressions for growth in each of these domains, a lower VABS-II or higher CBCL score at T1 was associated with growth on that domain (T1 communication [202 participants]: β = –0.07; OR, 0.94; 95% CI, 0.90-0.97; *P* < .01; T1 socialization [202 participants]: β = –0.10; OR, 0.91; 95% CI, 0.85-0.96, *P* < .01; T1 internalizing [178 participants]: β = 0.09; OR, 1.09; 95% CI, 1.04-1.14, *P* < .001; T1 externalizing [178 participants]: β = 0.10; OR, 1.10; 95% CI, 1.06-1.15; *P* < .001). The PLS-4 was associated with growth in socialization (β = 0.04; OR, 1.04; 95% CI, 1.00-1.07; *P* = .04). General family functioning was also associated with growth in internalizing (β = –1.03; OR, 0.36; 95% CI, 0.16-0.79; *P* = 0.01) and higher household income was associated with growth in externalizing (β = –0.80; OR, 0.45; 95% CI, 0.18-1.00; *P* = .03). Previous work on the Pathways data set indicated that SES, child age at diagnosis, and mother age at consent have been associated with differences in rates of attrition.^46^ Sensitivity analysis including these variables in the logistic regression analyses did not change any of our results (eTable 6 in the Supplement).

**Table 5.** T1 Attributes Associated With T2 Doing Well by the Growth Metric in Each of the 5 Domains

Characteristic	OR (95% CI)				
	VABS-II Communication	VABS-II Activities of daily living^a^	VABS-II Socialization	CBCL Internalizing	CBCL Externalizing
T1 instrument score^b^	0.94 (0.90-0.97)^c^	0.91 (0.86-0.97)	0.91 (0.85-0.96)^c^	1.09 (1.04-1.14)^c^	1.10 (1.06-1.15)^c^
SES^d^	1.08 (0.51-2.29)	0.5 (0.20-1.23)	0.79 (0.34-1.86)	0.99 (0.50-1.97)	0.45 (0.22-0.91)^c^
Sex	0.59 (0.22-1.59)	2.09 (0.43-10.13)	1.6 (0.46-5.54)	0.61 (0.24-1.54)	1.28 (0.47-3.51)
Merrill Palmer	1.02 (0.99-1.04)	1.01 (0.98-1.04)	1 (0.97-1.03)	0.98 (0.96-1.01)	1.00 (0.97-1.02)
PLS-4	0.99 (0.95-1.03)	1.01 (0.97-1.05)	1.04 (1.00-1.07)^c^	1.01 (0.98-1.04)	1.00 (0.97-1.03)
Ways of coping	0.42 (0.13-1.31)	0.74 (0.16-3.34)	0.36 (0.09-1.45)	1.46 (0.51-4.18)	0.41 (0.14-1.23)
General family functioning	0.81 (0.34-1.89)	1.07 (0.36-3.15)	0.66 (0.25-1.75)	0.36 (0.16-0.79)^c^	0.43 (0.18-1.00)

Abbreviations: CBCL, Child Behavior Checklist; OR, odds ratio; PLS-4, Preschool Language Scale, Fourth Edition; SES, socioeconomic status; T1, samples assessed between ages 2 and 4.9 years; T2, samples assessed in middle childhood; VABS-II, Vineland Adaptive Behavior Scales–Second Edition.

^a^ The omnibus test for this model was not significant and the results on variables associated with this outcome should not be interpreted (eTable 5 in the Supplement).

^b^ T1 instrument scores represent the score on the instrument at T1 in the domain being analyzed (ie, in the analysis on VABS-II Communication outcome at T2, the instrument score VABS-II Communication at T1 is included in analysis).

^c^ *P* < .05.

^d^ Estimated self-reported income stratified at $80 000, with 0 = <$80 000 per year household income, and 1 = ≥$80 000 per year household income.

## Discussion

Our objective was to estimate the prevalence and attributes of doing well among children with ASD in middle childhood by applying Taylor’s^19^ framework of growth and proficiency. Our results suggest that doing well by either metric of growth or proficiency in at least 1 of the 5 developmental health domains was common (approximately 80%); 20% of children were doing well in 4 or more of the 5 domains evaluated. Children who did well according to the proficiency criterion were not necessarily those who did well according to the growth criterion. Doing well by either metric on measures of adaptive functioning or emotional health was not associated with scoring below the ASD cutoff on the ADOS and the SRS. The variable most associated with doing well was the initial score in that outcome domain at T1. Additional attributes of doing well were domain and metric specific, but in general included both child-specific characteristics, such as language, and contextual characteristics, such as household income and family functioning (controlling for baseline score in the outcome domain). Cognitive ability (at least as measured by the MPR) was not a significant factor in any outcome metric.

If different developmental domains in ASD have their own trajectories,^2^ it follows that doing well would be a multivariate construct. Given a measurement framework that incorporates multiple domains and metrics of growth and proficiency, it was encouraging to see that many children were doing well at age 8 to 10 years on at least a subset of domains. This provides support for incorporating a strengths-based approach to complement a deficit-based needs assessment in the ongoing care of children with ASD. A strengths-based perspective would support a more tailored and flexible approach to developing interventions, an approach that takes account of needs across a range of domains while at the same time making use of particular strengths that can be mobilized to improve the child-environment “fit.”^47^

It is perhaps not surprising that those with lower baseline scores on adaptive functioning (or more difficulties with emotional health) were more likely to show growth on those domains. Those with better adaptive functioning and emotional health were more likely to reach proficiency on those domains later on. These findings emphasize the potential importance of targeting early interventions to the domains considered key to doing well later in childhood. Interventions targeted only to symptoms of autism, or indeed cognitive ability, may not influence later outcomes in adaptive functioning or emotional health.^10^

The finding that family functioning is an important factor in several aspects of doing well suggests a new area of research focusing on the community and societal factors, whereby a well-functioning family might be able to improve outcomes for a child with ASD. Although we know that both proximal (ie, family functioning) and distal (socioeconomic disparities) contextual factors can influence typical child development, little research on psychosocial mediators and moderators of outcomes has been done in ASD. Our data support the need for such enquiries. One potential hypothesis to pursue is the possibility that higher income and good family functioning allow a family to access more resources or to apply developmentally appropriate and evidence-based interventions with greater fidelity. A research program embedded in a health services framework would be better positioned to address this issue in finer detail, especially given the results of the meta-analysis from Rogers et al^10^ of early interventions.

Because doing well is not a unitary construct, supporting children to do well will take a multipronged effort to address child, family, community, and societal factors. Systematically addressing the full breadth of modifiable factors during a child with ASD’s development may ensure that more children will do well over time.

### Strengths and Limitations

The strengths of our study include having a longitudinal design in an inception cohort with a large sample size, its assessment of multiple developmental domains, its assessment of the impact of missing data, and its use of models testing a range of child-related and contextual factors. By using criteria for doing well that were independent of ASD symptoms and of cognitive ability, we were able to uncouple the assessment of doing well from the persistence of diagnostic indicators, providing a strengths-based framework that can be applied to the broader continuum of children with ASD whether or not they meet a threshold for a diagnosis.

We are aware of several limitations to our analyses. First, our definition of “doing well” would be strengthened if we had data on its predictive validity (its stability). Second, attrition is a limitation because children with missing data differ to a small extent from those with complete data in some T1 characteristics. Third, a single informant (often the mother) provided these data. Fourth, the CBCL may not be sufficiently sensitive to measure emotional health in ASD and the decision that a score below the typical population average (ie, a T score less than 50) may not be a sufficiently valid proxy for emotional health.^33,34^ Fifth, while we used measurement tools based on population norms, we do not know the prevalence of demonstrated growth among typical children on each of the outcome domains. Sixth, the measurement model also suggests that within an instrument and within a metric, our definitions of doing well share informant and method variance and may also lead to some overlap in the results on attributes associated with outcomes via a single informant.

## Conclusions

This cohort study found that a substantial proportion of children with ASD were doing well by midchildhood according to at least 1 domain of developmental health. A crucial next step is for the ASD field—including young people with ASD, families, practitioners, researchers and policy makers—to come together and work toward consensus on what it means to do well in ASD at different developmental stages, as has been argued in recent articles.^18^ This discussion will include deciding which domains to incorporate, how elements of doing well should be operationalized within these domains, and whether any specific time points should be prioritized. Such consensus would support the development of a core outcome set for cohort studies, clinical trials in ASD,^48^ and routine clinical care. Some work in this area has already begun.^24^ An agreed-upon definition of doing well would also advance implementing a measurement-based care approach in ASD to monitor progress and to guide shared decision-making.^49^

Our findings on the importance of family income and family functioning remind us that disabilities do not exist apart from a social context. Children with ASD do well when, collectively, we create the social conditions for all people to participate and to experience their capabilities.^50^ Implementing this will require the active collaboration of all levels of government and policy makers as well as clinicians, researchers, family members, and people with ASD. We hope that the results of this study will encourage the ASD community writ large to also take a strengths-based approach to treatment planning as a way of supporting all children and families with ASD in doing well in every respect.

## Notes

Supplement. **eTable 1.** Missingness of T1 and T2 Characteristics of Participants **eTable 2.** Spearman's Correlation Coefficients Between Outcome Measures (CBCL and VABS-II) and Predictor Measures (Ways of Coping, General Family Function) That Were Combined **eTable 3.** Unweighted Cohen's Kappa for Agreement Between Binary Good Outcome Variables in the 5 Domains at T2 Within a Quantification Method at T2, and Agreement Between Quantification Methods **eTable 4.** Percent with Teacher-Completed SRS Scores ≤ 60 (Below Cut-off for ASD) or > 60 (Above Cut-off for ASD) for Each Outcome Metric Within Each Domain **eTable 5.** Omnibus Tests for Logistic Regression Models and Hosmer-Lemeshow for Suitability of Logistic Regression for Growth and Proficiency Metrics Across All Domains **eTable 6.** Sensitivity Analysis to Control for Variables Associated With Attrition in the Larger Pathways Study of T1 Variables Associated With T2 Metrics Doing Well in Each of the Five Domains (Omnibus α = 0.005) **eFigure.** Flow Chart of 5 Data Points in the Larger Pathways in ASD Study
