# Error in Byline

**DOI:** 10.1001/jamanetworkopen.2021.11245

**Published:** 2021-04-22

In the Original Investigation titled “Association of Child and Family Attributes With Outcomes in Children With Autism,”^1^ published March 29, 2021, there were errors in the spelling of 2 author names in the byline. Authors listed should have been Connor M. Kerns and Tracy Vaillancourt. This article has been corrected.
