# Supplementary material for: Association of Child and Family Attributes With Outcomes in Children With Autism
Source: JAMA Netw Open. 2021 Mar 29;4(3):e212530. doi: 10.1001/jamanetworkopen.2021.2530 (PMC8008283; doi:10.1001/jamanetworkopen.2021.2530)
Supplement: Supplement. — eTable 1. Missingness of T1 and T2 Characteristics of Participants eTable 2. Spearman's Correlation Coefficients Between Outcome Measures (CBCL and VABS-II) and Predictor Measures (Ways of Coping, General Family Function) That Were Combined eTable 3. Unweighted Cohen's Kappa for Agreement Between Binary Good Outcome Variables in the 5 Domains at T2 Within a Quantification Method at T2, and Agreement Between Quantification Methods eTable 4. Percent with Teacher-Completed SRS Scores ≤ 60 (Below Cut-off for ASD) or > 60 (Above Cut-off for ASD) for Each Outcome Metric Within Each Domain eTable 5. Omnibus Tests for Logistic Regression Models and Hosmer-Lemeshow for Suitability of Logistic Regression for Growth and Proficiency Metrics Across All Domains eTable 6. Sensitivity Analysis to Control for Variables Associated With Attrition in the Larger Pathways Study of T1 Variables Associated With T2 Metrics Doing Well in Each of the Five Domains (Omnibus α = 0.005) eFigure. Flow Chart of 5 Data Points in the Larger Pathways in ASD Study [file jamanetwopen-e212530-s001.pdf]

Supplemental Online Content

Szatmari P, Cost KT, Duku E, et al. Association of child and family attributes with outcomes in children with autism. *JAMA Netw Open*. 2021;4(3):e212530. doi:10.1001/jamanetworkopen.2021.2530

eTable 1. Missingness of T1 and T2 Characteristics of Participants

eTable 2. Spearman's Correlation Coefficients Between Outcome Measures (CBCL and VABS-II) and Predictor Measures (Ways of Coping, General Family Function) That Were Combined

eTable 3. Unweighted Cohen's Kappa for Agreement Between Binary Good Outcome Variables in the 5 Domains at T2 Within a Quantification Method at T2, and Agreement Between Quantification Methods

eTable 4. Percent with Teacher-Completed SRS Scores ≤ 60 (Below Cut-off for ASD) or > 60 (Above Cut-off for ASD) for Each Outcome Metric Within Each Domain

eTable 5. Omnibus Tests for Logistic Regression Models and Hosmer-Lemeshow for Suitability of Logistic Regression for Growth and Proficiency Metrics Across All Domains

eTable 6. Sensitivity Analysis to Control for Variables Associated With Attrition in the Larger Pathways Study of T1 Variables Associated With T2 Metrics Doing Well in Each of the Five Domains (Omnibus $\alpha = 0.005$)

eFigure. Flow Chart of 5 Data Points in the Larger Pathways in ASD Study

This supplemental material has been provided by the authors to give readers additional information about their work.

© 2021 Szatmari P et al. *JAMA Network Open.*

eTable 1. Missingness of T1 and T2 Characteristics of Participants. Significance tests are between participants with T1 data only and participants T1 & T2 data with independent t-tests for continuous data and Pearson's chi-square for categorical data. * p < .05; ** p < .01

	participants with T1 data only		*participants with T1 and T2 data*		
	**n**	**mean (SD)**	**n**	**mean (SD)**	**p**
**VABS communication ***	*129*	*70.50 (15.17)*	*272*	*74.59 (16.26)*	0.02*
**VABS activities of daily living**	129	76.50 (11.82)	272	76.08 (11.42)	0.73
**VABS social**	129	72.32 (9.05)	272	72.20 (9.12)	0.91
**CBCL internalising**	105	59.96 (9.22)	255	60.29 (9.25)	0.79
**CBCL externalising**	105	55.08 (10.16)	255	56.52 (10.44)	0.22
**Merrill-Palmer**	102	53.57 (25.90)	237	58.51 (25.85)	0.09
**PLS-4 ***	*122*	*62.08 (18.16)*	*262*	*66.74 (19.50)*	0.02*
**Parent disengaged emotion-focused coping**	103	0.02 (1.10)	262	0.00 (0.96)	0.89
**General Family Functioning**	109	1.77 (0.52)	260	(1.75 (0.47)	0.75
	**n**	**%**	**n**	**%**	**p**
**Bachelors' or more education attained by PMK**	112	42.9	260	41.6	0.76
**Bachelors' or more education attained by partner**	95	36.9	248	36.7	0.99
**Married or common law ****	*111*	*80.2*	*262*	*92.4*	< 0.01**
**Full-time employment of PMK**	112	31.3	263	28.9	0.60
**Full-time employments of partner**	96	83.3	251	83.3	0.94
**Self-report household income > $80,000 per year**	109	33.0	256	39.8	0.23
**European ethnic/cultural heritage of PMK**	111	78.4	263	71.5	0.17
**European ethnic/cultural heritage of partner**	96	79.2	252	73.8	0.34

© 2021 Szatmari P et al. *JAMA Network Open.*

eTable 2. Spearman's Correlation Coefficients Between Outcome Measures (CBCL and VABS-II) and Predictor Measures (Ways of Coping, General Family Function) That Were Combined.

CBCL Internalising		n	r
	between 8.74 and 10.76 years	143	0.75**
CBCL Externalising			
	between 8.74 and 10.76 years	143	0.69**
VABS-II Communication			
	between 8.74 and 10.76 years	199	0.69**
VABS-II Activities of Daily Living			
	between 8.74 and 10.76 years	198	0.87**
VABS-II Socialisation			
	between 8.74 and 10.76 years	197	0.82**
Ways of Coping: Disengaged, emotion-focused			
	between 3.41 and 4.02 years	254	0.60**
	between 3.41 and 4.53 years	236	0.60**
	between 4.02 and 4.53 years	212	0.71**
General Family Functioning			
	between 3.41 and 4.02 years	259	0.62**
	between 3.41 and 4.53 years	249	0.56**
	between 4.02 and 4.53 years	212	0.75**

** p< 0.001; r: Spearman's rho; VABS-II: Vineland Adaptive Behavior Scales, Second Edition; CBCL Child Behavior Checklist

© 2021 Szatmari P et al. *JAMA Network Open.*

eTable 3. Unweighted Cohen's Kappa for agreement between binary good outcome variables in the 5 domains at T2 within a quantification method at T2, and agreement between quantification methods.

	Within T2 Proficiency				Within Growth				Between T2 Proficiency and Growth
**Domains**	**1**	**2**	**3**	**4**	**1**	**2**	**3**	**4**	
1 VABS-II Communication									0.29 (0.18-0.40)
2 VABS-II Daily Living Skills	0.57* (0.46-0.66)				0.30 (0.17-0.44)				0.35 (0.22-0.48)
3 VABS-II Socialisation	0.50* (0.40-0.61)	0.43* (0.32-0.57)			0.35 (0.22-0.49)	0.43* (0.28-0.59)			0.38 (0.24-0.52)
4 CBCL Internalising	0.12 (-0.01-0.25)	0.10 (-0.03-0.23)	0.20 (0.08-0.33)		0.09 (-0.04-0.22)	0.04 (-0.07-0.15)	0.08 (-0.04-0.20)		0.55 (0.44-0.67)
5 CBCL Externalising	0.29 (0.16-0.41)	0.26 (0.14-0.37)	0.22 (0.12-033)	0.48* (0.37-0.59)	0.20 (0.06-0.33)	0.04 (-0.08-0.15)	0.18 (0.05-0.31)	0.45* (0.33-0.57)	0.41 (0.30-0.53)

© 2021 Szatmari P et al. *JAMA Network Open.*

**eTable 4**. Percent with Teacher-completed SRS scores ≤ 60 (below cut-off for ASD) or > 60 (above cut-off for ASD) for each outcome metric within each domain.							
		**n**	**% Teacher SRS ≤ 60 (95%CI)**	**% Teacher SRS > 60 (95%CI)**	**n**	**% Teacher SRS ≤ 60 (95%CI)**	**% Teacher SRS > 60 (95%CI)**
**Growth**		**Growth**			**No Growth**		
	VABS Communication	24	45.8 (26.2-66.8)	54.2 (33.2-73.8)	115	54.8 (45.3-64.0)	45.2 (36.0-54.7)
	VABS Socialisation	12	25.0 (6.7-57.2)	75.0 (42.8-93.3)	127	55.9 (46.8-64.6)	44.1 (35.4-53.2)
	VABS Activities of Daily Living	12	33.3 (11.3-64.6)	66.7 (35.4-88.7)	127	55.1 (46.1-63.9)	44.9 (36.1-53.9)
	CBCL Internalising	40	52.5 (36.3-68.2)	47.5 (31.8-63.7)	82	51.2 (40.0-62.3)	48.8 (37.7-60.0)
	CBCL Externalising	37	40.5 (25.2-57.8)	59.5 (42.2-74.8)	85	56.5 (45.3-67.1)	43.5 (32.9-54.7)
**Proficiency**		**Proficient**			**Not Proficient**		
	VABS Communication	50	30.0 (18.3-44.8)	70.0 (55.2-81.7)	89	66.3 (55.4-75.8)	33.7 (24.2-44.6)
	VABS Socialisation	28	25.0 (11.4-45.2)	75.0 (54.8-88.6)	111	60.4 (50.6-69.4)	39.6 (30.6-49.4)
	VABS Activities of Daily Living	34	23.5 (11.4-41.6)	76.5 (58.4-88.6)	105	62.9 (52.8-71.9)	37.1 (28.1-47.2)
	CBCL Internalising	47	46.8 (32.4-61.8)	53.2 (38.2-67.6)	75	54.7 (42.8-66.1)	45.3 (33.9-57.2)
	CBCL Externalising	60	40.0 (27.8-53.5)	60.0 (46.5-72.2)	62	62.9 (49.7-74.6)	37.1 (25.4-50.3)

© 2021 Szatmari P et al. *JAMA Network Open.*

**eTable 5.** Omnibus tests for logistic regression models and Hosmer-Lemeshow for suitability of logistic regression for growth and proficiency metrics across all domains.												
	**Proficiency**						**Growth**					
	**Omnibus test of the model**			**Hosmer-Lemeshow test of logistic regression suitability**			**Omnibus test of the model**			**Hosmer-Lemeshow test of logistic regression suitability**		
	**χ2**	**df**	**p**	**χ2**	**df**	**p**	**χ2**	**df**	**p**	**χ2**	**df**	**p**
**VABS-II Communication**	70.14	7	<0.005*	12.56	8	0.13	27.57	7	<0.005**	8.08	8	0.43
**VABS-II Activities of Daily Living**	42.29	7	<0.005*	6.85	8	0.55	17.15	7	0.02	2.94	8	0.94
**VABS-II Socialization**	47.61	7	<0.005*	8.73	8	0.37	22.73	7	<0.005**	7.21	8	0.51
**CBCL Internalising**	19.35	7	0.01	2.17	8	0.98	24.32	7	<0.005**	12.83	8	0.12
**CBCL Externalising**	44.89	7	<0.005*	6.99	8	0.54	33.84	7	<0.005**	5.41	8	0.71

© 2021 Szatmari P et al. *JAMA Network Open.*

eTable 6. Sensitivity analysis to control for variables associated with attrition in the larger Pathways study of T1 variables associated with T2 metrics doing well in each of the five domains (omnibus α = 0.005).

	PROFICIENCY					GROWTH				
**VABS-II Communication**	**χ2**	**df**	**p**			**χ2**	**df**	**p**		
OMNIBUS test of the full model	71.46	9	***< 0.005***			27.99	9	***0.001***		
Hosmer-Lemeshow	8.44	8	0.39			12.06	8	0.15		
	**B**	**OR**	**LL 95% CI**	**UL 95% CI**	**p**	**B**	**OR**	**LL 95% CI**	**UL 95% CI**	**p**
Child age at diagnosis	-0.02	0.98	0.94	1.03	0.40	-0.01	0.99	0.94	1.04	0.62
Age of mother at time of consent	0.00	1.00	1.00	1.01	0.38	0.00	1.00	1.00	1.01	0.63
SES	-0.14	0.87	0.43	1.76	0.70	0.15	1.17	0.54	2.54	0.70
T1 Instrument Score	0.05	1.05	1.01	1.09	***0.017****	-0.07	0.93	0.90	0.97	***0.001****
Child biological sex	-0.05	0.95	0.33	2.73	0.93	-0.47	0.63	0.23	1.70	0.36
Merrill Palmer	0.02	1.02	1.00	1.05	0.10	0.02	1.02	0.99	1.05	0.16
PLS-4	0.02	1.02	0.98	1.05	0.33	-0.01	0.99	0.96	1.03	0.74
Ways of Coping	-0.97	0.38	0.13	1.10	0.08	-0.88	0.41	0.13	1.35	0.14
General Family Functioning	-0.18	0.84	0.38	1.85	0.66	-0.23	0.80	0.34	1.87	0.60
**VABS-II Activities of Daily Living**	**χ2**	**df**	**p**			**χ2**	**df**	**p**		
OMNIBUS test of the full model	45.09	9	***< 0.005***			17.48	9	0.04		
Hosmer-Lemeshow	4.56	8	0.80			3.44	8	0.90		
	**B**	**OR**	**LL 95% CI**	**UL 95% CI**	**p**	**B**	**OR**	**LL 95% CI**	**UL 95% CI**	**p**
Child age at diagnosis	-0.04	0.96	0.92	1.01	0.11	0.00	1.00	0.94	1.06	0.92
Age of mother at time of consent	0.00	1.00	0.99	1.01	0.75	0.00	1.00	0.99	1.01	0.59
SES	-0.39	0.68	0.33	1.39	0.29	-0.73	0.48	0.19	1.25	0.13
T1 Instrument Score	0.06	1.07	1.03	1.11	***0.001****	-0.09	0.91	0.86	0.97	0.00

© 2021 Szatmari P et al. *JAMA Network Open.*

	Child biological sex	0.68	1.97	0.66	5.90	0.22	0.70	2.02	0.41	9.92	0.39
	Merrill Palmer	0.00	1.00	0.98	1.03	0.92	0.01	1.01	0.98	1.04	0.55
	PLS-4	0.03	1.03	1.00	1.06	0.05	0.01	1.01	0.97	1.05	0.79
	Ways of Coping	-0.79	0.45	0.15	1.40	0.17	-0.34	0.71	0.16	3.26	0.66
	General Family Functioning	-0.56	0.57	0.25	1.31	0.19	0.07	1.07	0.36	3.19	0.90
**VABS-II Socialisation**		**χ2**	**df**	**p**			**χ2**	**df**	**p**		
	OMNIBUS test of the full model	51.17	9	***< 0.005*** **			27.25	9	***0.001*** **		
	Hosmer-Lemeshow	11.57	8	0.17			9.6	8	0.29		
		**B**	**OR**	**LL 95% CI**	**UL 95% CI**	**p**	**B**	**OR**	**LL 95% CI**	**UL 95% CI**	**p**
	Child age at diagnosis	-0.05	0.96	0.91	1.01	0.10	-0.06	0.94	0.88	1.00	***0.039****
	Age of mother at time of consent	0.00	1.00	1.00	1.01	0.28	0.00	1.00	0.99	1.01	0.83
	SES	0.47	1.60	0.71	3.60	0.26	-0.08	0.92	0.38	2.25	0.85
	T1 Instrument Score	0.10	1.10	1.05	1.16	***< 0.001****	-0.12	0.89	0.83	0.95	***< 0.001****
	Child biological sex	0.42	1.53	0.46	5.10	0.49	0.73	2.07	0.55	7.85	0.29
	Merrill Palmer	0.03	1.03	1.00	1.06	0.07	0.01	1.01	0.98	1.04	0.50
	PLS-4	0.01	1.01	0.98	1.04	0.63	0.04	1.04	1.00	1.08	***0.044****
	Ways of Coping	-0.45	0.64	0.19	2.09	0.46	-1.17	0.31	0.07	1.33	0.12
	General Family Functioning	-1.03	0.36	0.14	0.91	***0.031****	-0.51	0.60	0.22	1.63	0.32
**CBCL Internalising**		**χ2**	**df**	**p**			**χ2**	**df**	**p**		
	OMNIBUS test of the full model	19.66	9	0.02			24.41	9	***0.004*** **		
	Hosmer-Lemeshow	5.54	8	0.7			7.97	8	0.44		
		**B**	**OR**	**LL 95% CI**	**UL 95% CI**	**p**	**B**	**OR**	**LL 95% CI**	**UL 95% CI**	**p**
	Child age at diagnosis	-0.01	0.99	0.95	1.04	0.79	0.00	1.00	0.96	1.05	0.97
	Age of mother at time of consent	0.00	1.00	0.99	1.00	0.67	0.00	1.00	0.99	1.01	0.79
	SES	-0.21	0.81	0.41	1.61	0.55	-0.02	0.99	0.49	1.98	0.97
	T1 Instrument Score	-0.05	0.96	0.92	1.00	0.03	0.09	1.09	1.04	1.14	***< 0.001****
	Child biological sex	-0.46	0.63	0.25	1.59	0.33	-0.51	0.60	0.24	1.53	0.29

© 2021 Szatmari P et al. *JAMA Network Open.*

	Merrill Palmer	-0.01	0.99	0.96	1.01	0.25	-0.02	0.98	0.96	1.01	0.17
	PLS-4	0.01	1.01	0.98	1.04	0.65	0.01	1.01	0.98	1.04	0.60
	Ways of Coping	-0.04	0.96	0.34	2.68	0.93	0.37	1.44	0.50	4.17	0.50
	General Family Functioning	-0.85	0.43	0.20	0.94	0.03	-1.02	0.36	0.16	0.80	***0.012****
**CBCL Externalising**		**χ2**	**df**	**p**			**χ2**	**df**	**p**		
	OMNIBUS test of the full model	45.93	9	***< 0.005*****			34.47	9	***<0.005*****		
	Hosmer-Lemeshow	6.22	8	0.62			5.17	8	0.74		
		**B**	**OR**	**LL 95% CI**	**UL 95% CI**	**p**	**B**	**OR**	**LL 95% CI**	**UL 95% CI**	**p**
	Child age at diagnosis	0.01	1.01	0.97	1.06	0.57	0.01	1.01	0.96	1.06	0.79
	Age of mother at time of consent	0.00	1.00	0.99	1.00	0.35	0.00	1.00	0.99	1.00	0.43
	SES	-0.69	0.50	0.25	1.02	0.06	-0.86	0.42	0.20	0.88	***0.021****
	T1 Instrument Score	-0.07	0.94	0.90	0.97	***0.001****	0.10	1.10	1.06	1.15	***< 0.001****
	Child biological sex	0.55	1.73	0.66	4.56	0.26	0.23	1.26	0.46	3.46	0.66
	Merrill Palmer	-0.02	0.98	0.96	1.01	0.17	-0.01	0.99	0.97	1.02	0.64
	PLS-4	0.02	1.02	0.99	1.06	0.17	0.00	1.00	0.97	1.03	0.90
	Ways of Coping	-0.98	0.38	0.13	1.09	0.07	-0.91	0.40	0.13	1.22	0.11
	General Family Functioning	-0.98	0.37	0.17	0.85	***0.018****	-0.82	0.44	0.19	1.03	0.06

© 2021 Szatmari P et al. *JAMA Network Open.*

eFigure. Flow Chart of the 5 Data Points in the Larger Pathways in ASD study. Data from the first three Pathways data points comprise the current study T1 time point in these analyses. Data from the second two data points comprise the study T2 time point in these analyses. When needed, we took an average of scores across the data points to minimize missing data and to provide a more stable assessment of outcome scores than relying on single time points alone. To justify the approach of using mean scores in place of single scores, we ran preliminary analysis (not shown) to consider whether measures were on average significantly correlated across time points using Pearson's correlations. In all cases, measures were significantly correlated across time points (all p's < 0.001, al r's > 0.49).

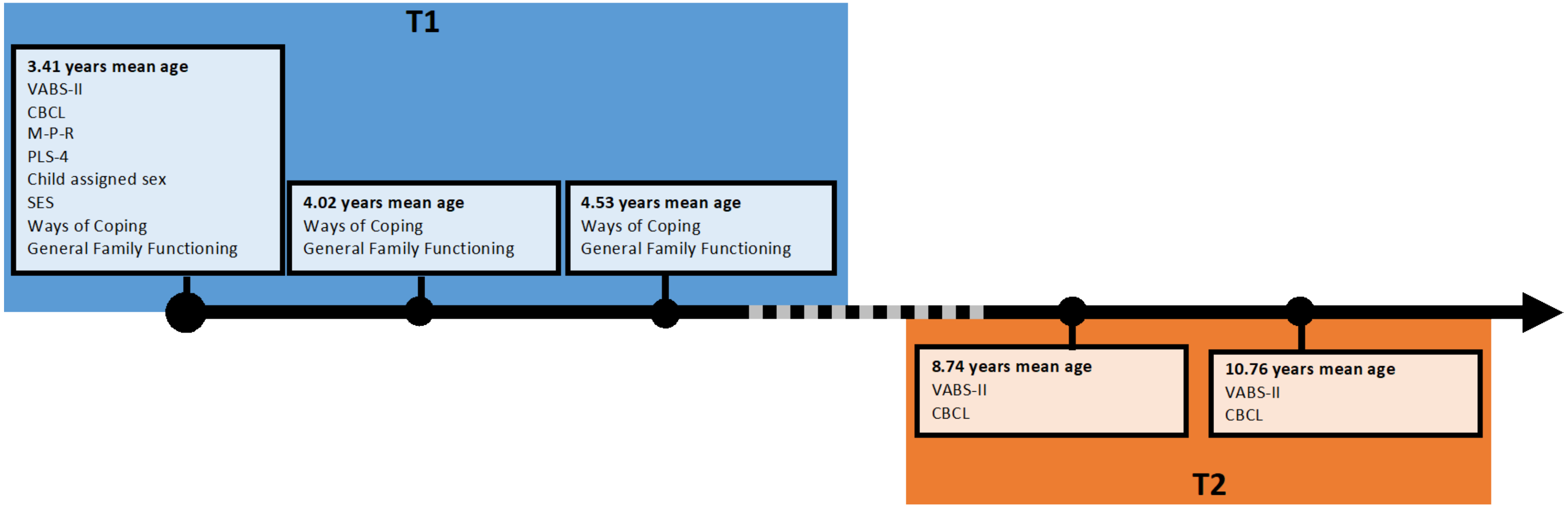

© 2021 Szatmari P et al. *JAMA Network Open.*
